# Evaluation of MRI-derived surrogate signals to model respiratory motion

**DOI:** 10.1088/2057-1976/ab944c

**Published:** 2020-06-12

**Authors:** Elena H Tran, Björn Eiben, Andreas Wetscherek, Uwe Oelfke, Gustav Meedt, David J Hawkes, Jamie R McClelland

**Affiliations:** 1 Centre for Medical Image Computing, Department of Medical Physics and Biomedical Engineering, University College London, London, United Kingdom; 2 Joint Department of Physics, The Institute of Cancer Research and The Royal Marsden NHS Foundation Trust, London, United Kingdom; 3 Elekta, Medical Intelligence Medizintechnik GmbH, Schwabmünchen, Germany; elena.tran.16@ucl.ac.uk

**Keywords:** respiratory surrogate signals, internal signals, image-derived signals, surrogate-driven motion model, respiratory motion model, MR-Linac, MRI-guided radiotherapy

## Abstract

An MR-Linac can provide motion information of tumour and organs-at-risk before, during, and after beam delivery. However, MR imaging cannot provide real-time high-quality volumetric images which capture breath-to-breath variability of respiratory motion. Surrogate-driven motion models relate the motion of the internal anatomy to surrogate signals, thus can estimate the 3D internal motion from these signals. Internal surrogate signals based on patient anatomy can be extracted from 2D cine-MR images, which can be acquired on an MR-Linac during treatment, to build and drive motion models. In this paper we investigate different MRI-derived surrogate signals, including signals generated by applying principal component analysis to the image intensities, or control point displacements derived from deformable registration of the 2D cine-MR images. We assessed the suitability of the signals to build models that can estimate the motion of the internal anatomy, including sliding motion and breath-to-breath variability. We quantitatively evaluated the models by estimating the 2D motion in sagittal and coronal slices of 8 lung cancer patients, and comparing them to motion measurements obtained from image registration. For sagittal slices, using the first and second principal components on the control point displacements as surrogate signals resulted in the highest model accuracy, with a mean error over patients around 0.80 mm which was lower than the in-plane resolution. For coronal slices, all investigated signals except the skin signal produced mean errors over patients around 1 mm. These results demonstrate that surrogate signals derived from 2D cine-MR images, including those generated by applying principal component analysis to the image intensities or control point displacements, can accurately model the motion of the internal anatomy within a single sagittal or coronal slice. This implies the signals should also be suitable for modelling the 3D respiratory motion of the internal anatomy.

## 1. Introduction

An MR-Linac is an MR-image guided radiotherapy (MR-IGRT) system which enables imaging of a patient’s internal anatomy in real-time before, during, and after radiotherapy treatment. Many prototypes have been proposed over the last decade and some of them have become commercially available (Raaymakers *et al* 2009, Fallone 2014, Keall *et al* 2014, Low *et al* 2016). MR-IGRT systems may improve tumour control and decrease toxicity to the surrounding healthy tissues especially for moving targets, allowing hypo-fractionated or dose-escalated radiotherapy treatments (Bainbridge *et al* 2017, Pathmanathan *et al* 2018).

Respiratory motion can be a major problem for lung cancer radiotherapy as it introduces uncertainty in the delivered dose. In particular, it can lead to the tumour receiving less dose and/or the healthy tissues receiving more dose than planned. Breathing motion can vary within a single treatment fraction (intra-fraction) due to irregular breathing, and can change between fractions (inter-fraction), for instance, when there are anatomical and physiological changes during the course of radiotherapy (Keall *et al* 2006).

2D sagittal cine-MR images are available for on-line tumour imaging during treatment with MR-IGRT systems. They have been used to guide gated stereotactic delivery to treat tumours and nodes in the lung (Fischer-Valuck *et al* 2017, van Sörnsen de Koste *et al* 2018), and could guide tumour trailing (Fast *et al* 2019) or tracked treatments (Crijns *et al* 2012, Menten *et al* 2016) using dynamic multi-leaf collimators. This may be sufficient to account for the tumour motion in the case of negligible through-plane motion. However, some lung tumours exhibit asymmetric 3D trajectories showing hysteresis (Seppenwoolde *et al* 2002). Furthermore, the motion of organs-at-risk (OARs) cannot be accounted for, unless they lie in the same plane used to image the tumour motion. Therefore, real-time volumetric information should ideally be used to guide treatment delivery. In addition, knowledge of the motion of the full 3D anatomy is required to accurately estimate the delivered dose. This would facilitate implementation of inter-fraction and intra-fraction adaptation of radiotherapy treatments based on the dose that was actually delivered (Kontaxis *et al* 2015).

There have been many 4D-MRI methods proposed in recent years, as described in detail in recent review papers of MRI for radiotherapy applications (Stemkens *et al* 2018, Paganelli *et al* 2018b). Due to the inherent trade-off between temporal resolution, spatial resolution and field-of-view in MR imaging, most of the proposed methods are respiratory-correlated 4D-MRI. These techniques use data acquired from different respiratory cycles to produce retrospectively-sorted 3D volumes at different respiratory states (Cai *et al* 2011, Deng *et al* 2016, Li *et al* 2017, Mickevicius and Paulson 2017, Han *et al* 2017, van de Lindt *et al* 2018, Lee *et al* 2019, van Kesteren *et al* 2019). Real-time guidance information cannot be provided since the images are not available until all data have been acquired. Other research groups have implemented time-resolved 4D-MRI techniques which acquire volumetric images fast enough to sample respiratory motion, but spatial resolution and image quality are limited compared to respiratory-correlated 4D-MRI (Dinkel *et al* 2009, Yang *et al* 2015, Yuan *et al* 2019), and current time-resolved 4D-MRI images are not suitable for providing real-time guidance information.

Respiratory motion models could provide a solution to these problems, and a detailed review of these models can be found in McClelland *et al* (2013). Different research groups have proposed respiratory motion models which estimated the 3D motion from 2D cine-MR images, acquired using a 2D image navigator, for MRI-guided radiotherapy (Stemkens *et al* 2016, Harris *et al* 2016) or PET-MR applications (King *et al* 2012, Fayad *et al* 2012). These models were generated by applying principal component analysis (PCA) to the deformation fields derived from the registration of 3D MRI volumes. To obtain time-resolved 3D motion estimates from the 2D cine-MR images, the PC weights were optimized by maximizing the similarity between the 3D reference volume deformed according to the PCA-based motion model and the current 2D cine-MR image(s).

However, all these approaches present limitations related to the 3D MRI volumes used to build the PCA-model. For Fayad *et al* (2012) and King *et al* (2012) the dynamic 3D MRI volumes were characterized by poor signal-to-noise ratio of the structures inside the lung. For Stemkens *et al* (2016) and Harris *et al* (2016) the 3D MRI volumes were retrospectively-sorted, thus they suffered from sorting artefact which can affect the derived deformation fields used to build the model. Paganelli *et al* (2019) compared the models proposed by Fayad *et al* (2012) and Stemkens *et al* (2016) with other approaches generating time-resolved volumetric MRI for MR-guided radiotherapy (Seregni *et al* 2017, Paganelli *et al* 2018a, Garau *et al* 2019) by using the XCAT computerized anthropomorphic phantom. This study showed that the models proposed by Fayad *et al* (2012) and Stemkens *et al* (2016) were not able to accurately model the 3D motion from 2D cine-MR images when the motion seen during 2D cine-MR acquisition differed from the average cycle represented by the 3D MRI volumes used to build the PCA-model. Furthermore, all investigated approaches were based on one or all 10 respiratory phases of an ideal pre-treatment 4D-MRI without image artefacts which was generated using the XCAT, and would not be available for real patient datasets.

Surrogate-driven motion models relate the motion of the internal anatomy to surrogate signal(s). The surrogate signals are measured instead of the full motion of interest which is estimated by the models. To overcome the lack of 3D images suitable to build motion models, we proposed a different approach in McClelland *et al* (2017), where we built surrogate-driven motion models by unifying the image registration and motion model fitting into a single optimization, enabling the motion model to be fitted directly to all of the dynamic image data simultaneously. Importantly, this meant it was not necessary to sort the dynamic data into respiratory-correlated 3D volumes before fitting the models, rather, a 3D motion model could be fitted directly to the unsorted data, e.g. the individual slices from a multi-slice MR acquisition. Promising qualitative results were obtained from sagittal multi-slice MR images of both lungs, imaged with overlapping slices to enable a motion-compensated super resolution reconstruction (McClelland *et al* 2017). However, the accuracy of these models was not quantitatively assessed as the true 3D motion is not known and cannot be estimated independently of the motion models.

MR-based motion models have also been proposed for a wide range of applications, including PET-MR (Baumgartner *et al* 2014, Manber *et al* 2016, Küstner *et al* 2017), MR-guided high intensity focused ultrasound and radiotherapy (Baumgartner *et al* 2017). However, none of these models used surrogate signals derived from 2D cine-MR images to drive the motion model. Indeed, using surrogate signals from 2D cine-MR images may be seen as a disadvantage for some applications, as the acquisition of these images can effectively double the acquisition time. Contrarily, on the MR-Linac it is desirable to acquire the 2D cine-MR images during treatment delivery to monitor the tumour and guide gated or tracked treatments, so surrogate signals that can be derived from the 2D cine-MR images, and can drive a motion model, are ideal for this application.

Different methods of acquiring surrogate signals have been proposed. However, many external devices producing external signals are not suitable for use on an MR-Linac Instead, an MR-Linac gives the opportunity to extract internal surrogate signals from the 2D cine-MR images acquired during treatment.

Several MRI-derived surrogate signals have been proposed in the literature to drive respiratory motion models, or to retrospectively sort MR images into respiratory-correlated 4D-MRI volumes. The most widely used surrogate signal is derived from a 1D MR navigator which usually includes the interface between lung and liver to extract the diaphragm motion (King *et al* 2011, Stemkens *et al* 2015, Li *et al* 2017). Some studies exploited 2D cine-MR images to extract a surrogate signal from a region of interest, such as the body area or skin surface (Cai *et al* 2011, Mcglashan and King 2011). Other studies proposed to apply the 2D Fourier transform on each frame of a 2D cine-MRI series, and generate a respiratory signal from the phase components of low-frequency elements in the Fourier space (Cai *et al* 2015, Hui *et al* 2016). Surrogate signals can be generated using information available from the MR acquisition itself. For instance, self-gated techniques can derive a respiratory signal from the center of the k-space of a 3D radial stack-of-stars acquisition (Buerger *et al* 2012, Rank *et al* 2017, Mickevicius and Paulson 2017). Andreychenko *et al* (2018) used the thermal noise variance of the receiver radio-frequency coils, obtained from the raw k-space data, to generate surrogate signals to model respiratory motion. None of these studies have compared different methods to generate surrogate signals from 2D cine-MR images for building and driving respiratory motion models. Therefore, the aim of this work is to investigate different methods to generate surrogate signals for respiratory motion modelling from 2D cine-MR images, similar to those that can be acquired on an MR-Linac during treatment.

**Figure 1.**
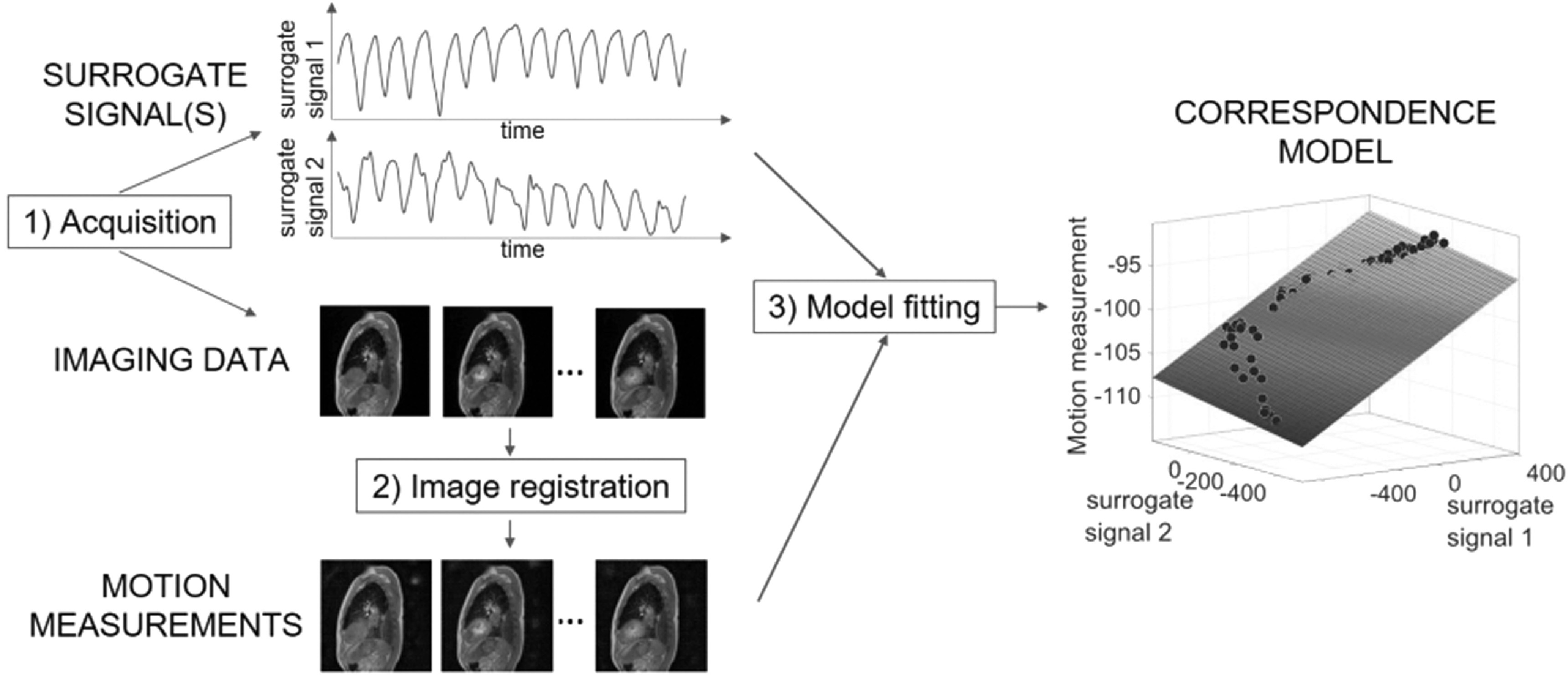
Typical approach to build a surrogate-driven motion model. 1) Acquisition of surrogate signal(s) and imaging data, 2) image registration of imaging data to obtain motion measurements, 3) model fitting to obtain a correspondence model.

In this study we want to compare different MRI-derived surrogate signals by quantitatively assessing the accuracy of the corresponding surrogate-driven motion models. This is difficult with our approach presented in McClelland *et al* (2017) because it unifies the image registration and model fitting into a single optimization, and independent motion measurements are not available to compare to the motion estimated by the model. Therefore, we decided to employ the more typical approach to build motion models, which is shown schematically in figure 1, where the image registration is performed prior to fitting the motion models and can provide an independent estimate of the motion with which to quantitatively assess the models. The typical approach includes the following steps: 1) simultaneous acquisition of surrogate and imaging data, 2) image registration performed on the imaging data to obtain measurements of the motion of interest, 3) model fitting to obtain a correspondence model which describes the mathematical relationship between the surrogate signals and the motion of interest. By choosing appropriate surrogate signals and correspondence models it is possible to model both intra-cycle (hysteresis) and inter-cycle (breath-to-breath) variability of respiratory motion (McClelland *et al* 2013). Once the model is built, it takes the surrogate signal(s) as input and returns the current motion estimate as output.

As already discussed, it is challenging to generate accurate 3D motion measurements from 3D MRI volumes: time-resolved 4D-MRI is not well suited for this purpose due to the limited image quality and spatial resolution resulting from the high temporal resolution. Respiratory-correlated 4D-MRI volumes are also not ideal for this purpose. Most methods assume reproducible breathing, so they cannot be used to model or assess inter-cycle variation, and will often suffer from sorting artefacts. Some methods cover many respiratory cycles and can estimate inter-cycle variability (Von Siebenthal *et al* 2007, Celicanin *et al* 2015). However, they require very long acquisition times, and each volume is still generated from data acquired at different time points from different breath cycles, so does not represent a unique point in time and hence may not necessarily give a good representation of the true 3D motion and its variability.

For this reason, in this study we built and evaluated 2D motion models of the patients’ anatomy within a 2D slice using the typical approach. This enabled us to estimate both the intra- and inter-cycle variability in the respiratory motion, and assess the ability of the different surrogate signals to model this variability. Both sagittal and coronal slices were used to assess the ability to model motion in all 3 spatial directions. The 2D motion at each time point was estimated using a deformable image registration algorithm that can preserve sliding motion (Eiben *et al* 2018), and used to build and evaluate the models.

We investigated linear correspondence models relating the motion to two or three surrogate signals. We also analyzed the effect of the training data size on the model accuracy, and the inter-patient variability of the accuracy of the different models.

These 2D models were not intended for clinical use themselves as they only provided 2D motion information. Instead, they represented a means of quantitatively assessing the different surrogate signals and informing the choice of signal(s) for future work that utilizes our approach in McClelland *et al* (2017) to build 3D motion models for planning and guiding treatments on an MR-Linac.

## 2. Materials and methods

### 2.1. Image acquisition

This study included 8 lung cancer patients after written informed consent. Patient characteristics are reported in table 1. We retrospectively used datasets acquired for a previous study (Fast *et al* 2017) using a 1.5T MR scanner (MAGNETOM Aera, Siemens Healthcare, Erlangen, Germany) with the patients scanned in free-breathing. For each patient two different datasets of 2D cine-MR images, referred to as the sagittal and coronal datasets, were acquired for approximately one minute each, using a spoiled gradient echo sequence with the following parameters: TR = 3.2 s (TR = 3.4 s for patient 3 only), TE = 1.37 s, image resolution = 1.98 × 1.98 ×10 mm^3^, acquisition matrix = 192 × 171, image matrix = 192 × 192.

**Table 1.** Patient characteristics. TNM staging was performed according to the AJCC recommendations (Edge and Compton 2010). The range of motion of the center-of-mass (COM) of the tumour was not computed for patient 5 who had the primary tumour previously resected, and for the coronal datasets excluded from the study (see section 2.2.2).

Patient	Sex	Age	Pathology	TNM	Tumour position	Tumour COM range of motion (mm)			
						Sag dataset		Cor dataset	
						SI	AP	SI	LR
1	M	76	NSCLC	T4N0M0	Left hilar	6.6	3.4	6.0	3.8
2	M	50	SCLC	T4N3M0	Left lower lobe	7.5	3.5	7.3	2.3
3	F	79	NSCLC	T2N0M0	Left upper lobe	22.8	6.2	26.9	3.7
4	M	70	NSCLC	T4N2M0	Right upper lobe	1.0	0.7	1.0	1.7
5	M	57	NSCLC	T0N2M0	(*)	—	—	—	—
6	M	60	SCLC	T4N3M0	Right middle lobe	22.8	5.7	—	—
7	M	68	NSCLC	T4N0M1a	Left lower lobe	1.6	0.9	—	—
8	F	73	NSCLC	T2aN2M0	Right lower lobe	18.7	4.1	—	—

*Abbreviations*: (N)SCLC = (non) small cell lung cancer. Sag = sagittal. Cor = coronal. (*) = Left paratracheal lymph node involvement.

For each dataset, the images were alternately acquired from two fixed slice locations, positioned to image the tumour (or lymph node in case of recurrence after resection). Images from one fixed slice location are called surrogate images, and they were used to generate the surrogate signals. Sagittal orientation was chosen for the surrogate images because it typically captures the predominant respiratory motion, which tends to be in the superior-inferior (SI) and anterior-posterior (AP) directions (Seppenwoolde *et al* 2002). Images from the other fixed slice location are called motion images, and they were used to estimate the 2D motion of the anatomy within a single slice. For each sagittal dataset the motion images had sagittal orientation, and they were acquired from a slice location adjacent to the location of the surrogate slice. For each coronal dataset the motion images had coronal orientation, and they were acquired from a slice intersecting the surrogate slice through the lesion. While through-slice motion impacts coronal slices more than sagittal slices, coronal motion slices were included in this study to investigate how well the signals extracted from a sagittal surrogate slice were able to model both the left-right (LR) motion and the motion of anatomical structures which were further away from the surrogate slice.

For all patients except patient 3 sagittal datasets comprised 120 surrogate and 120 motion images, while coronal datasets consisted of 180 surrogate and 180 motion images. For patient 3 the sagittal dataset included 100 surrogate and 100 motion images while the coronal dataset consisted of 150 surrogate and 150 motion images.

### 2.2. Pre-processing

Except where specified otherwise, all pre-processing tasks described below, and all processing tasks for surrogate signal generation, model fitting and evaluation, were performed using MATLAB (version 2017a, The Mathworks, Natick, MA).

#### 2.2.1. Surrogate images

Pre-processing steps were carried out on the surrogate images for all datasets to remove potential confounding factors which could affect the comparison between the different surrogate signals in estimating the respiratory motion. Firstly, the images at the start of each acquisition had a lower acquisition rate due to the acquisition of reference lines for the GRAPPA reconstruction (Deshmane *et al* 2012), and were discarded. The frame rate of the remaining images was ∼1.9 fps for the sagittal datasets and ∼2.9 fps for the coronal datasets (respectively ∼1.8 fps and ∼2.7 fps for patient 3 only). Secondly, if a slice is excited repeatedly before the longitudinal magnetization has completely recovered, the mean image intensity decreases over time due to saturation effects before reaching a stationary condition. Therefore, we used a simple threshold on the mean image intensity to exclude images acquired before the steady state condition had been reached. After the described pre-processing, the number of discarded surrogate images varied between 2 and 5 for all datasets.

#### 2.2.2. Motion images and generation of motion measurements

Wrap-around artefacts in the coronal images of the coronal datasets for patient 2 and patient 5 were removed by cropping the images. For patient 6 three of the motion images from the sagittal dataset included a sudden and evident bulk motion of the whole body (including both in-plane and out-of-plane motion). These were excluded as the aim of this study was to assess the ability of the surrogates to model respiratory motion rather than bulk motion.

Deformable image registration was applied to the motion images of each dataset to obtain motion measurements of the internal anatomy. We used an extension of the open-source software NiftyReg^4^ ^4^
https://github.com/KCL-BMEIS/niftyreg
 which can account for sliding motion (Eiben *et al* 2018). NiftyReg is based on the fast free-form deformation algorithm with the cubic B-splines transformation model defined on a control point grid (Modat *et al* 2010). Full details of the modifications that allow for sliding motion can be found in Eiben *et al* (2018), and only a brief summary is given here. The source (moving) image is segmented into two regions that can move independently and hence slide past each other, with a separate transformation used for each region. An extra penalty term is introduced to penalize gaps and overlaps that occur between the two sliding regions.

**Figure 2.**
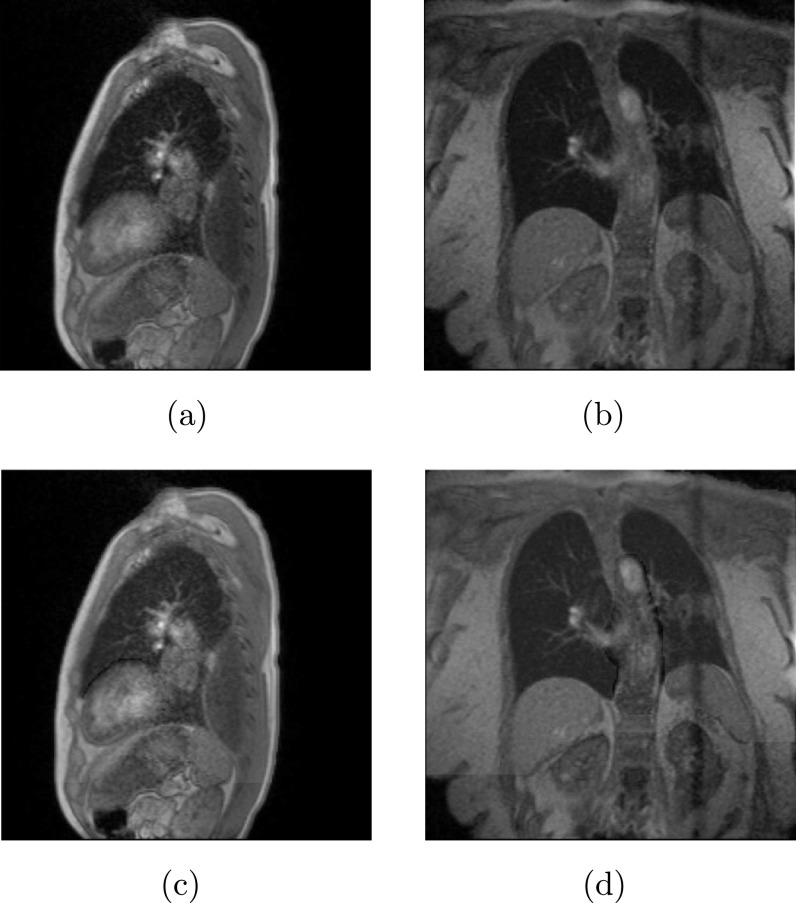
Examples of the segmentation required for the sliding registration, and identifying the two sliding regions in the source image for a sagittal dataset (a) and a coronal dataset (b). Examples of the evaluation mask used for the evaluation of the models for a sagittal dataset (c) and a coronal dataset (d). Patient 2 is imaged in (a) and (c), while patient 3 is imaged in (b) and (d). The band of reduced signal intensities in the coronal motion slice in (b) and (d) is caused by the reduced longitudinal magnetization recovery where the coronal motion slice intersects the interleaved sagittal surrogate slice.

For each dataset we selected an image at end-exhale position as the source image for all registrations, with the other images from the dataset being used as target images. We manually segmented the source image using ITK-Snap (version 3.6.0) so that one region included the lungs, mediastinum, and abdominal organs, which can slide past the chest wall during respiration. An example of the segmentation for a sagittal and a coronal dataset is shown in figures 2(a) and (b), respectively. We utilized the same registration parameters for all datasets, particularly, we used locally normalized cross-correlation (LNCC) as an image similarity measure (Cardoso *et al* 2013), and bending energy, linear energy, and gap-overlap penalty terms with weights of 0.001, 0.01, and 0.1 respectively. Three resolution levels were used, with control point grid spacings of 40, 20, and 10 mm. The registration results from the motion images were parameterized by the B-spline control point displacements (CPD_*m*_). Two full grids of control points were required, one for each of the sliding regions.

We visually assessed the registrations using colour overlays between the deformed source images and corresponding target images. The motion slice of three coronal datasets (from patients 6, 7, and 8) was located at the very back of the lung and included the whole spine. The registrations for these datasets failed because of the considerable noise present in the acquired images, which affected especially the lung. The results of the failed 2D registration for these cases do not represent realistic measurements of the internal motion, and should therefore not be used to build nor to assess the motion estimated by the models. Therefore, we excluded these datasets from the rest of the study.

As a result, 8 sagittal datasets and the remaining 5 coronal datasets were considered for the evaluation of the surrogate signals. Supplementary movies show an example of the registration results for a sagittal and a coronal dataset (1__reg__results__sag__patient2.mp4 and 2__reg__results__cor__patient3.mp4, respectively). In some cases the registrations produced unrealistic looking motion in the heart and major blood vessels due to blood flow causing large intensity changes, and in the abdomen due to digestive and other non-respiratory motion causing through-plane motion. As the aim of this study was to assess the ability of the surrogate signals to model respiratory motion, and the registration results in these regions did not represent respiratory motion, they were ignored when assessing the motion models. For all registration results we computed the Jacobian determinant (Brock *et al* 2017) which is a measure of the local volume change resulting from the registration. All Jacobian determinant values were positive indicating transformations which do not contain any folding (Brock *et al* 2017).

### 2.3. Generation of surrogate signals

We generated both local and global surrogate signals from the surrogate images for each dataset. Local surrogates were extracted by following the motion of local anatomical structures included in the images. Global surrogates were generated by exploiting information from the whole image (or anatomy) using principal component analysis (PCA). All signals were interpolated at the time points of the motion images by fitting a smooth spline function to the extracted surrogate signal data points. To avoid extrapolation of the surrogate signals, all motion images (and corresponding registration results) outside the interval time between the first and last surrogate images were discarded.

#### 2.3.1. Local surrogate signals

We used diaphragm and skin to generate local surrogate signals, because they are commonly used as surrogates for respiratory motion.

**Figure 3.**
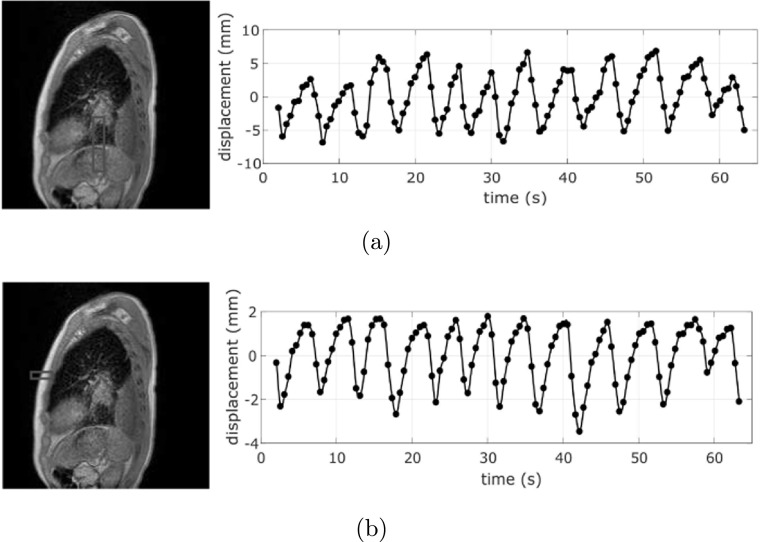
Example of interpolated local surrogate signals for the sagittal dataset of patient 2. The extracted surrogate signal values are indicated with the symbol (•). (a) Window used for diaphragm detection (left), and diaphragm signal (right), (b) window used for skin surface detection (left), and skin signal (right). The positive and negative peaks of the signals indicate the end-exhalation and end-inhalation, respectively.

(i) Diaphragm signal
  : The diaphragm signal was given by the SI displacement of the diaphragm relative to its average position expressed in mm. It was generated by manually identifying a point on the boundary between diaphragm and lung in the first surrogate image, and setting a rectangular window around it (20 or 50 pixels in SI direction, and 6, 10 or 20 pixels in AP direction, depending on the slope of the diaphragm and the presence of other anatomical structures). Then, we used an in-house algorithm to detect the vertical position of the diaphragm boundary with sub-pixel accuracy using just the part of the image within the window. An example of the window set around the diaphragm and the generated signal is reported in figure 3(a).
(ii) Skin signal
  : The skin signal was determined by the AP displacement of the skin surface relative to its average position expressed in mm. An example of the skin signal is reported in figure 3(b). To generate the signal, we manually selected a point on the skin surface of the chest in the first surrogate image, and we applied the same algorithm used for the diaphragm detection (using a rectangular window with 20 pixels in the AP direction, and 6 or 10 pixels in the SI direction, depending on the slope of the skin surface).

#### 2.3.2. Global surrogate signals

PCA is a linear dimensionality reduction technique which aims to preserve the variation in the original dataset (Jolliffe and Cadima 2016). It finds a set of new orthogonal coordinate axes, called principal components (PCs), from linear combinations of the original variables, such that the first PC accounts for as much variation in the data as possible, the second PC accounts for as much of the remaining variation as possible, and so on. This means that it is often possible to represent a large proportion of the variation in the original data using relatively few PCs. The coordinates of a data point projected on to the PCs are called the PC scores (or weights).

We investigated the PC scores of the first three PCs (i.e. PC1, PC2, PC3) on image intensities or B-spline control point displacements from the surrogate images (CPD_*s*_), as potential surrogate signals to build and drive motion models (Tran *et al* 2019a).

**Figure 4.**
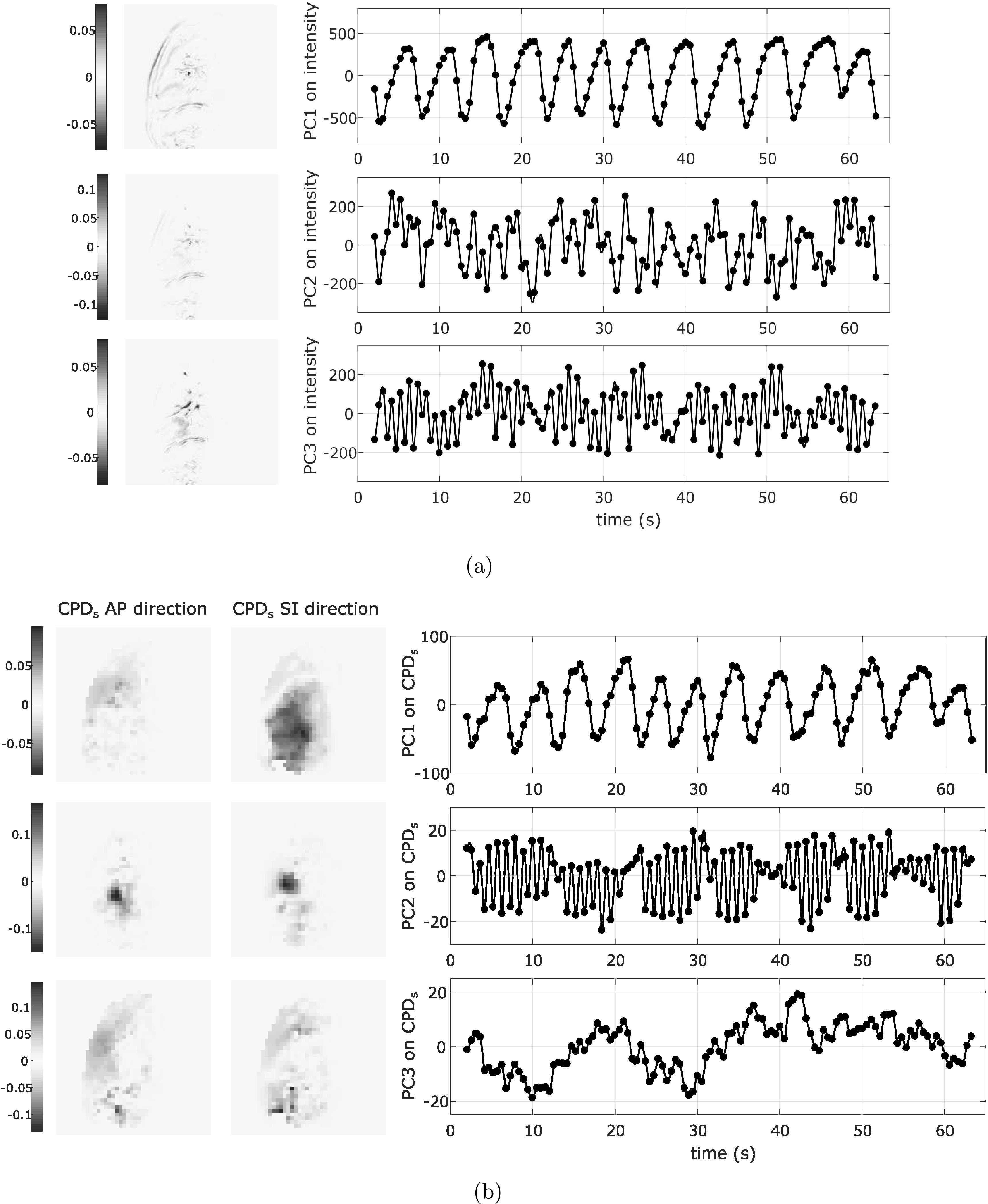
Example of interpolated global surrogate signals for the sagittal dataset of patient 2, with the surrogate images including the heart. The extracted surrogate values are indicated with the symbol (•). (a) Coefficient maps and signal for PC1, PC2, and PC3 on intensity, (b) coefficient maps relative to the CPD_*s*_ in AP (left) and SI (right) directions, and signal for PC1, PC2, and PC3 on CPD_*s*_.

(i) PCA on image intensities
  : PCA was applied to the image intensities of all pixels in the surrogate images. Figure 4(a) shows an example of the extracted signals and the corresponding PC coefficient maps, which illustrate the contribution of each pixel to the specific PC.
(ii) *PCA on CPD*_*s*_
  : Deformable image registration was performed on the surrogate images using NiftyReg (Modat *et al* 2010). We used locally normalized cross-correlation (LNCC) as an image similarity measure (Cardoso *et al* 2013), and the bending energy as regularization term with weight of 0.005. Three resolution levels were used with control point grid spacings of 20, 10, and 5 pixels. For each patient, the average position of the diaphragm was determined from the diaphragm signal generated using the first 30 images (covering 3 to 5 breath cycles). The reference image was chosen to be the one with the diaphragm closest to its average position. PCA was applied to the CPD_*s*_, excluding control points that were not within the patient’s body. For this purpose, a binary mask was generated from the reference image for each patient, using thresholding to exclude the background (and necessarily the low-intensity regions such as the lung), followed by the closing morphological operation to include the low-intensity body regions in the mask. Figure 4(b) shows an example of the extracted signals and the corresponding PC coefficient maps for each CPD_*s*_ in AP and SI directions.

### 2.4. Study design and experiments

Two or more surrogate signals are needed to drive a motion model which is able to model both intra-cycle and inter-cycle variation of respiratory motion (Mcglashan and King 2011, McClelland *et al* 2013). More signals can potentially model more variation but require more data to avoid over-fitting. Therefore, in this study we investigated linear correspondence models relating the motion to two (Equation (1)) or three (equation (2)) surrogate signals:

1 \begin{eqnarray*}{M}_{i}({s}_{1},{s}_{2})={c}_{2}{s}_{2}+{c}_{1}{s}_{1}+{c}_{0}\end{eqnarray*}

2 \begin{eqnarray*}{M}_{i}({s}_{1},{s}_{2},{s}_{3})={c}_{3}{s}_{3}+{c}_{2}{s}_{2}+{c}_{1}{s}_{1}+{c}_{0}\end{eqnarray*}

${M}_{i}$ is the *i*^*th*^ component of the CPD_*m*_ (i.e the displacement in the AP, SI, or LR direction for one control point) obtained from the registration of the motion images (see section 2.2.2), ${s}_{j}$ are the surrogate signals, and ${c}_{0}$, ${c}_{1}$, ${c}_{2}$, ${c}_{3}$ are the motion model parameters that were determined by performing an ordinary least squares fit to the data. Previous works have related the motion to a surrogate signal and its temporal derivative, rather than two different signals (McClelland *et al* 2013). Therefore, we investigated models that use signals and their temporal derivative as well as models that use independent signals. The different combinations of surrogate signals used for the motion models are given in table 2. A total of 7 different 2-signal models and 3 different 3-signal models were investigated.

**Table 2.** Different combinations of surrogate signals used for the surrogate-driven motion models.

	Surrogate signals
2-signal models	PC1 on CPD_*s*_, derivative
	PC1 on intensity, derivative
	Diaphragm, derivative
	Skin, derivative
	Diaphragm, skin
	PC1, PC2 on CPD_*s*_
	PC1, PC2 on intensity

3-signal models	Diaphragm, derivative, skin
	PC1, PC2, PC3 on CPD_*s*_
	PC1, PC2, PC3 on intensity

No motion model	None

**Figure 5.**
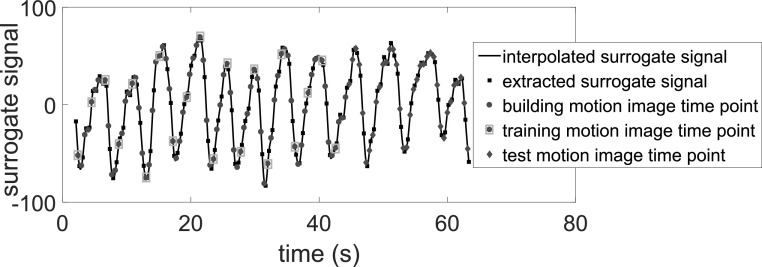
Example of the interpolated surrogate signal and the surrogate signal values (■) extracted from the surrogate images. Subdivision of the motion image time points into a building set (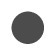), and a test set (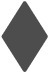) used to evaluate the models. The training set (indicated by a box around the data points) is a subset of the building set used to train the different surrogate-driven motion models.

As illustrated in figure 5, each dataset was divided into a building set, comprising the first 80 motion images, and a test set including the remaining motion images (between 20 and 100 images, see section 2.1).

The interpolated surrogate signals’ values of the test set were used as input for each motion model to yield motion estimates. The model estimated the CPD_*m*_ for both sliding regions, from which we calculated the deformation vector fields (DVF) defined at each pixel location (Eiben *et al* 2018). Then, we computed the deformation field error (DFE) defined as the L2-norm difference between the model estimated DVF and the DVF provided by image registration. To calculate statistical values for the DFE, we manually generated a binary mask for the source image of each dataset, referred to as the evaluation mask, using ITK-Snap. The evaluation mask included the patients’ body, but excluded all regions where the registration results were considered implausible due to through-plane motion or blood flow, as discussed in the section 2.2.2. An example of the evaluation mask for a sagittal and a coronal dataset are shown in figures 2(c) and (d), respectively. We manually segmented the tumour in the source image of each dataset to obtain a binary mask for the tumour region only. This was not possible for the sagittal and coronal datasets from patient 5 which did not include a primary tumour but the involved lymph node only (see table 1). To obtain the evaluation mask and tumour mask for each test time point, we warped the corresponding masks from the source image to each test motion image using the registration results. Mean and 95th percentile DFE were computed within the evaluation mask and within the tumour mask over all test motion images for each dataset. For both masks we also calculated the DFE with the estimated motion set to 0, i.e. corresponding to the case when no model is used, to quantify the amount of motion included in the test motion images.

#### 2.4.1. Effect of training set size on model accuracy

We investigated the effect of the number of training motion images used on the accuracy of the motion models. From the building set a subset of motion images, referred to as the training set, was used to train the different models. We built motion models using training sets of $n=20,19,18,...,6$ images. When using a training set of 20 images, every 4th image in the building set of 80 images was used (as shown in figure 5). The training images were evenly spaced in time over the building set to mimic the acquisition pattern that would be used for 3D data (although for 3D data the time between successive images would be much longer (Tran *et al* 2019b)). This enabled a 4-fold cross-validation to be performed, with training sets consisting of images [1, 5, .., 77], [2, 6, .., 78], [3, 7, .., 79], and [4, 8, .., 80] from the building set. When using smaller training sets, the earliest images were discarded and the images closest to the test set were retained for building the models, e.g. when using 19 training images the first training set consisted of the images [5, 9, .., 77].

All models were evaluated on the full test set available for that acquisition. The mean and 95th percentile DFE were averaged over the 4 iterations of cross-validation and over all 8 patients for the sagittal datasets, and over the 5, or 4, considered patients for the coronal datasets when using the evaluation mask, or tumour mask, respectively (see section 2.4).

#### 2.4.2. Inter-patient variability of model accuracy

We analyzed the inter-patient variability of the DFE for the different models to assess whether it is suitable to use the same surrogates for all patients, or whether different patients may benefit from using different surrogates. The DFE statistics were averaged over the 4 iterations of cross-validation for each patient individually. Due to the large number of models evaluated above, the inter-patient comparison was only performed for a fixed number of training images (determined from the results of the previous experiment).

## 3. Results

### 3.1. Effect of training set size on model accuracy

**Figure 6.**
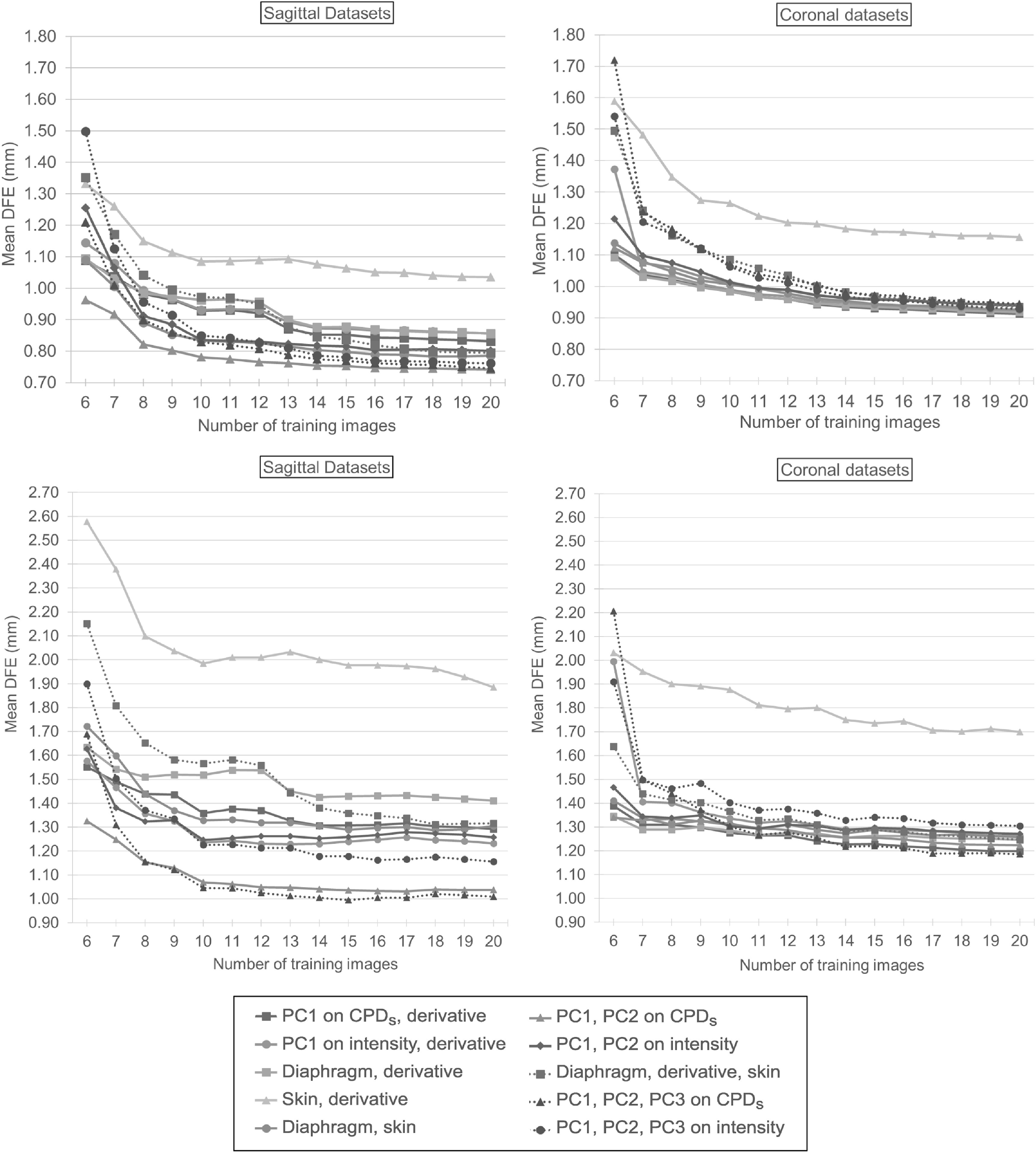
Mean DFE (in mm) for the different models as function of the number of training motion images for sagittal datasets (left) and coronal datasets (right). The different models are indicated by different lines: two-signal models are marked with a solid line, three-signal models are marked with a dotted line. Top: Mean DFE within the evaluation mask with the *y*-axis cut-off at 0.70 mm. When no model was used, the mean DFE was 1.90 mm for the sagittal datasets, and 2.30 mm for the coronal datasets. Bottom: Mean DFE within the tumour mask with the *y*-axis cut-off at 0.90 mm. When no model was used, the mean DFE was 3.21 mm for the sagittal datasets, and 3.00 mm for the coronal datasets.

**Figure 7.**
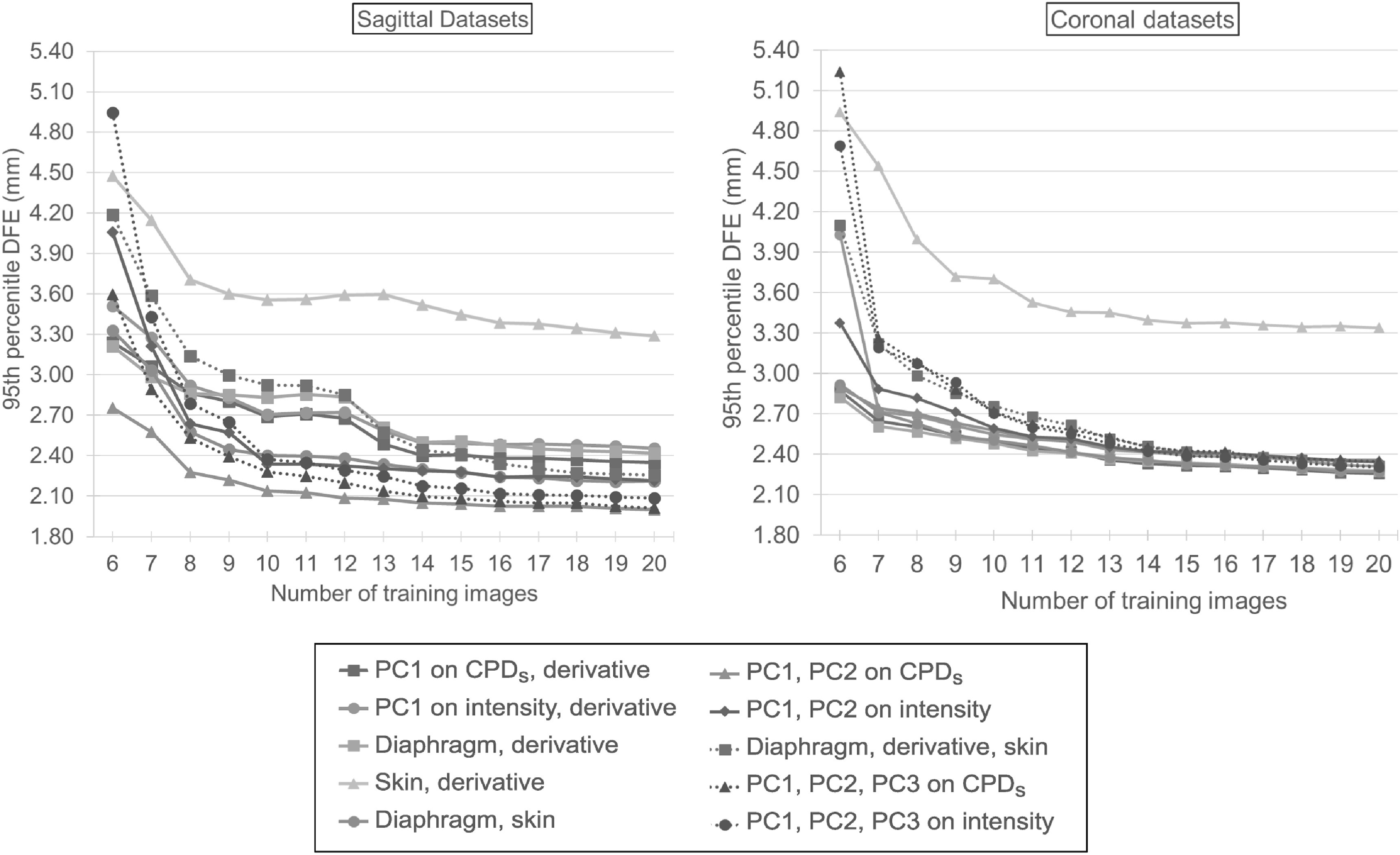
95th percentile DFE (in mm) within the evaluation mask for the different models as function of the number of training motion images for sagittal datasets (left) and coronal datasets (right). The different models are indicated by different lines: two-signal models are marked with a solid line, three-signal models are marked with a dotted line. The *y*-axis is cut-off at 1.80 mm. When no model was used, the 95th percentile DFE was 6.71 mm for the sagittal datasets, and 9.01 mm for the coronal datasets.

Figure 6 (top) and figure 7 show mean and 95th percentile DFE within the evaluation mask, respectively, measured in mm and averaged over patients for sagittal and coronal datasets. These statistics are reported as functions of the number of training motion images for the different models. The graphs show that all models substantially improved the DFE when compared to using no model. The accuracy of all models improved as more training images were used. The performance gains decreased with the amount of images used, and the performance of all models had approximately plateaued when using 20 images.

In general the different models had similar performance, especially on the coronal datasets, except for the model based on the skin signal and its derivative, which performed noticeably worse than the other models unless only 6 training images were used. The 3-signal models had worse results for coronal datasets when using smaller number of images, and they did not show an improvement over 2-signal models when using more images. For the sagittal datasets the models driven by the derivative as one of the signals showed a sudden increase of the DFEs when less than 13 images were used. This behaviour was not shown by the models driven by independent signals.

For the sagittal datasets the most accurate model was driven by PC1 and PC2 on CPD_*s*_. These surrogates produced mean (95th percentile) DFEs between 0.74 mm and 0.96 mm (2.00 mm and 2.75 mm), compared to a mean (95th percentile) DFE of 1.90 mm (6.71 mm) when no model was used. For the coronal datasets the most accurate models were driven by either the diaphragm signal or PC1 on CPD_*s*_ in combination with their derivative. For these surrogates the mean (95th percentile) DFE was around 1.00 mm (2.50 mm), compared to a mean (95th percentile) DFE of 2.30 mm (9.01 mm) when no model was used. All other 2-signal models, except for the skin signal and the case of 6 training images, yielded mean (95th percentile) DFEs which differed from the most accurate models by 0.07 mm (0.30 mm) or less.

Figure 6 (bottom) shows the mean DFEs within the tumour mask as function of the number of training motion images for the different models relative to the sagittal and coronal datasets. Overall for the tumour region we obtained results comparable to the case of the evaluation mask. For the sagittal datasets the main difference was that the 3-signal model driven by PC1, PC2 and PC3 on CPD_*s*_ produced the lowest mean DFEs of around 1.00 mm with 10 or more images, compared to a mean DFE of 3.21 mm obtained without any model. However, the difference between the 3-signal model and the 2-signal model driven by the PCs from the CPD_*s*_ was negligible when using 8 or more training images, and the 3-signal model produced higher DFEs than the 2-signal model when using fewer training images, as observed for the evaluation mask. For the coronal datasets, except for the skin signal and its derivative and the case of 6 training images, all models produced mean DFEs between 1.20 mm and 1.50 mm compared to a mean DFE of 3.00 mm obtained without any model.

### 3.2. Inter-patient variability of model accuracy

The effect of the training set size on the mean DFE within the evaluation mask for the different models, analyzed for each patient individually, is reported in the supplementary data (Supplementary figure 3 for sagittal datasets, and Supplementary figure 4 for coronal datasets available online at stacks.iop.org/BPEX/6/045015/mmedia). When using just 6 training images, for 3 patients some models produced mean DFEs which were higher than the mean DFE obtained without a model. For instance, this applied to the 3-signal models for both sagittal and coronal datasets.

Based on the results shown in figures 6 and 7, the inter-patient comparison was performed for the case of 10 training images, because this represented a reasonable choice regarding the trade-off between model accuracy and using fewer numbers of training images. Tables 3 and 4 report the mean DFEs within the evaluation mask for each patient relative to the sagittal and coronal datasets, respectively. These results are expressed in mm and compared to the mean and standard deviation values calculated over all patients.

**Table 3.** Mean DFEs within the evaluation mask (in mm) for each patient. The different surrogate-driven motion models were evaluated using sagittal datasets and 10 training motion images. Mean and standard deviation (std) values over patients are reported in the last two columns. Best performing models are highlighted in bold.

	Patient								
Surrogate signals	1	2	3	4	5	6	7	8	mean	std
PC1 on CPD_*s*_, derivative	0.78	0.77	0.76	0.84	0.70	2.31	0.59	0.69	0.93	0.56
PC1 on intensity, derivative	**0.76**	0.77	0.77	0.84	0.72	2.26	0.63	0.71	0.93	0.54
Diaphragm, derivative	0.77	0.77	0.75	0.82	0.70	2.45	0.62	0.81	0.96	0.60
Skin, derivative	0.84	0.80	0.99	1.02	0.97	2.45	0.61	1.01	1.08	0.57
Diaphragm, skin	0.80	**0.74**	0.71	0.90	**0.67**	1.50	0.65	0.69	0.83	0.28
PC1, PC2 on CPD_*s*_	0.81	0.76	**0.70**	**0.74**	0.70	**1.30**	**0.58**	0.64	**0.78**	**0.22**
PC1, PC2 on intensity	**0.76**	0.79	0.79	0.89	0.81	1.34	**0.58**	0.74	0.84	**0.22**
Diaphragm, derivative, skin	0.80	0.76	0.76	1.04	0.74	2.26	0.68	0.73	0.97	0.53
PC1, PC2, PC3 on CPD_*s*_	0.85	0.76	0.72	0.79	0.82	1.49	0.59	**0.61**	0.83	0.28
PC1, PC2, PC3 on intensity	0.79	0.80	0.71	0.82	0.74	1.70	0.60	0.64	0.85	0.35

No motion model	1.75	1.26	2.30	1.34	1.58	3.80	1.03	2.18	1.90	0.88

As previously observed, the most accurate model for the sagittal datasets was driven by PC1 and PC2 on CPD_*s*_. As shown in table 3, this model produced the lowest mean DFE for 4 out of 8 patients, and mean DFEs which were very close to the best model for all the 4 remaining patients. It also yielded the lowest standard deviation of 0.22 mm, whereas all models driven by the derivative as one of the surrogates were characterized by standard deviation values above 0.50 mm. This was largely due to patient 6, for whom all the models using derivatives produced mean DFEs larger than 2.2 mm.

**Figure 8.**
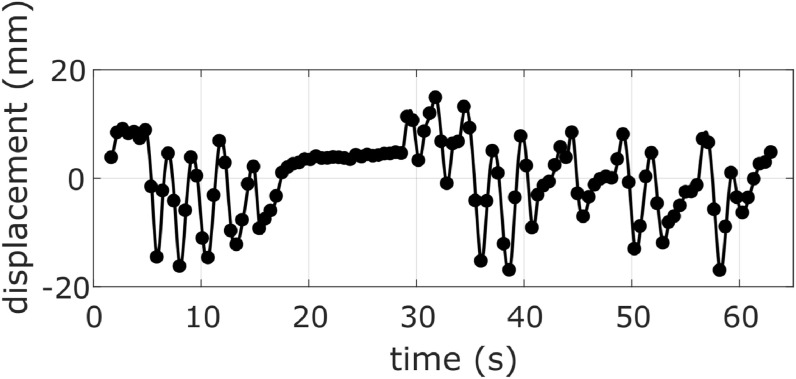
Diaphragm signal illustrating the irregular breathing pattern, with a temporary breath-hold, for patient 6.

Patient 6 was characterized by a complex motion of the diaphragm and irregular breathing pattern, including a temporary breath-hold, as shown by the diaphragm signal in figure 8. For this patient using the derivative as one of the surrogate signals (except for the skin signal and its derivative) produced a considerable increase of the mean DFEs when the training set included less than 13 images (see Supplementary figure 3). The trend of the DFE curves obtained for patient 6 was reflected in the overall results within the evaluation mask for the sagittal datasets shown in figure 6 (top) and figure 7. A supplementary movie shows the estimated motion obtained when using the diaphragm signal and its derivative, or PC1 and PC2 on CPD_*s*_ (5__model__results__PC1__2__onCPD__diaphragm__patient6.mp4). While PC1 and PC2 on CPD_*s*_ were able to model the internal motion well, including sliding motion, the model driven by the diaphragm signal and its derivative produced notable errors for the diaphragm, the tumour and the vessels in the lung, indicated by coloured areas in the colour overlay image in the supplementary movie.

For the coronal datasets table 4 shows that all models, except for the one driven by the skin signal and its derivative, produced mean DFEs around 1 mm for each patient. These results were obtained regardless of the amount of motion included in the original test images and quantified by the mean DFE obtained when no model was used, which reached values up to 2.81 mm (for patient 2). The standard deviation values for these models were below 0.1 mm, which was less than $20 \% $ of the standard deviation obtained when no model was used (0.52 mm).

**Table 4.** Mean DFEs within the evaluation mask (in mm) for each patient. The different surrogate-driven motion models were evaluated using coronal datasets and 10 training motion images. Mean and standard deviation (std) values over patients are reported in the last two columns. Best performing models are highlighted in bold.

	Patient						
Surrogate signals	1	2	3	4	5	mean	std
PC1 on CPD_*s*_, derivative	0.97	1.01	0.98	0.97	0.99	0.99	**0.02**
PC1 on intensity, derivative	**0.94**	**1.00**	1.06	**0.95**	0.99	0.99	0.05
Diaphragm, derivative	1.00	1.06	**0.92**	0.96	0.99	**0.98**	0.05
Skin, derivative	0.97	1.08	1.55	1.08	1.65	1.26	0.31
Diaphragm, skin	1.05	1.10	0.95	0.96	0.98	1.01	0.06
PC1, PC2 on CPD_*s*_	1.08	1.02	1.04	0.96	**0.96**	1.01	0.05
PC1, PC2 on intensity	1.06	**1.00**	0.99	0.99	1.03	1.01	0.03
Diaphragm, derivative, skin	1.03	1.17	0.98	1.14	1.10	1.09	0.08
PC1, PC2, PC3 on CPD_*s*_	1.10	1.09	0.96	1.09	1.11	1.07	0.06
PC1, PC2, PC3 on intensity	1.03	1.10	1.02	1.05	1.12	1.06	0.05

No motion model	1.98	2.81	2.35	1.59	2.75	2.30	0.52

The results for the tumour region are reported for each patient individually in the supplementary data (Supplementary tables S1 and S2). The tables S1 and S2 in the supplementary file show the mean DFEs averaged over all test time points for sagittal and coronal datasets, respectively. They also include the mean DFEs at the test time point corresponding to the deepest end-inhale, considered as the worst-case scenario. The models produced larger improvements over no model for those patients (3, 6 and 8) who had a tumour characterized by considerable sliding motion against the chest wall. For those patients, the most accurate model driven by PC1, PC2 and PC3 on CPD_*s*_ produced mean DFEs of around 1 mm, which was below the pixel size, although the mean DFEs without any model and averaged over all time points (at the deepest end-inhale) reached values of 7.21 mm (21.23 mm).

## 4. Discussion

In this paper we compared different methods to extract surrogate signals from 2D cine-MR images. We investigated both global and local surrogate signals to model the internal motion for lung cancer patients.

The global signals were generated by applying principal component analysis (PCA) to the 2D displacements of the control points (CPD_*s*_), or image intensities.

The CPD_*s*_ principal components (PCs) were derived directly from the motion of the internal anatomy, parameterized by the registration results of the surrogate images. The sliding-preserving registrations were not used, so that the processing was fully automated and as fast as possible. The image intensities PCs were affected by the intensity changes due to the motion, but also blood flow. We found that the CPD_*s*_ PCs accounted for much more variation than the image intensities PCs. The percentages of the total variance of the original datasets, averaged over all sagittal and coronal datasets, were 70 ± 13%, 7 ± 4%, 3 ± 1% for PC1, PC2, PC3 on CPD_*s*_, and 33 ± 6%, 9 ± 4%, 5 ± 1% for PC1, PC2, PC3 on intensity.

As shown in figure 4, when the surrogate images included the heart, the CPD_*s*_ PCs differentiated respiratory and cardiac motion, while the intensity PCs mixed-up respiratory and cardiac induced intensity changes. However, the image intensities PCs are faster to calculate than the CPD_*s*_ PCs since no registration is required.

Previous works have used PCA to generate surrogate signals to drive respiratory motion models, but these have applied PCA to raw PET data (Manber *et al* 2016), or the thermal noise variance obtained from the raw k-space data (Andreychenko *et al* 2018). To the best of our knowledge, PCA has not been used before to generate surrogate signals from 2D cine-MR images to build and drive motion models, as proposed in this study.

For comparison, we also investigated local signals which are commonly used as surrogates for the respiratory motion, namely the diaphragm and the skin surface.

The motion of the skin surface is used by optical tracking systems, which are commercially available to monitor respiratory motion (Vedam *et al* 2003, Li *et al* 2012), but are not suitable for use on an MR-Linac It should be noted that we generated the skin signal from a region on the chest. A signal from the abdomen may have better correlated with the internal motion (Koch *et al* 2004) and led to better results for the skin and derivative model. However, we expected the chest signal to contain more complementary information to the diaphragm signal, and hence produce lower errors when combined with the diaphragm signal. Indeed, the model using both the skin and diaphragm signals did produce one of the lowest DFEs.

In theory, if more surrogate signals are used then more of the variability in the motion can be modelled, however, more data is required to robustly fit the models, which could lead to over-fitting. The results presented here showed that the models with 3 signals performed worse than the models with just 2 signals, especially when using fewer training images on the coronal datasets.

As shown in figure 6 (top) and figure 7, the models based on derivative signals were less accurate on the sagittal datasets than those that used independent signals, but this was due to a single patient (6) who had a very irregular breathing pattern that included a temporary breath-hold. Nevertheless, derivative based models have been widely used in the literature, and produced DFEs comparable to the models based on independent signals for all other patients. Future work will need to assess exactly why the derivative based models performed poorly for this patient, and determine how common such cases are before concluding that models based on independent signals should be preferred to models using derivative based signals. However, this result did suggest that derivative based signals may not be suitable for some individuals.

Overall this study found that the PC1 and PC2 on CPD_*s*_ signals gave the best results when used as surrogates for modelling the patients’ internal motion. However, the mean DFEs of all of the signals investigated was at least approximately half for the evaluation mask (or a third for the tumour) of the mean DFEs obtained when no model was used, with the exception of the skin signal and derivative model (and as previously noted, this may have been improved if a region on the abdomen had been used instead of the chest).

In the sagittal datasets the motion and surrogate slices were adjacent to each other, thus only the motion of structures close to the surrogate slice was modelled and evaluated in the sagittal orientation. This was due to the retrospective nature of our study (see section 2.1). However, the coronal datasets tested the ability of the surrogate signals to model motion further away from the surrogate slice, as well as motion in the left-right direction.

The larger errors for the coronal datasets compared to the sagittal datasets may be due to the fact that the coronal images contained anatomy that was more distant to the surrogate slices and/or included more motion (as seen from the larger DFE for the evaluation mask when no model was used). The different conclusions drawn for the accuracy of the investigated models for the sagittal and coronal datasets (see Results section 3, figures 6 and 7) may be due to the relatively small number of patients and/or the absence of very irregular breathing cases for the coronal datasets.

The peak-to-peak diaphragm motion amplitude, averaged over patients, was 16.5 mm and 27.0 mm for sagittal and coronal datasets, respectively, demonstrating that the datasets did contain a typical amount of respiratory motion. When no model was used, the mean DFE values obtained for the evaluation mask, equal to 1.90 mm for sagittal datasets and 2.30 mm for coronal datasets, were one order of magnitude lower than the values of the peak-to-peak diaphragm motion amplitude. It should be noted that the DFE values were averaged over all pixels in the evaluation mask, including anatomical structures with little or no motion. Furthermore, they were averaged over all time points, including those where the anatomy was close to the reference position (end-exhale).

Same considerations applied to the tumour motion and corresponding mean DFE values. For patients 3, 6 and 8, who had a tumour with considerable sliding motion against the chest wall, the range of the tumour COM motion amplitude in SI direction ranged from 18.7 mm to 26.9 mm, while the mean DFEs when using no model ranged from 4.89 mm to 7.21 mm. When no model was used, the mean DFEs for the deepest end-inhale time point, representing the worst-case scenario, had the same order of magnitude of the range of the tumour COM motion, and ranged between 13.87 mm and 21.23 mm.

For patient 4 all of the models produced mean DFEs within the tumour region which were higher than the mean DFE without any model when 10 training images or less were used. This could be explained by the fact that this patient had a big and stationary tumour with homogeneous intensity (average 2D GTV of 86.1 cm^2^ with COM motion range of 1.0 mm in SI direction), and when using few training images the models fitted the noise present in the acquired images within the tumour.

The aim of this study was to quantitatively compare the ability of the different surrogate signals to model the motion of the internal anatomy, and not to produce models that were directly intended for clinical use themselves. Therefore, we decided to use 2D cine-MR images rather than 4D-MRI to build and assess the models, as discussed in the Introduction 1. Furthermore, we visually assessed the motion slice registration results for this study using the colour overlay between the acquired images and the registration results, and we excluded regions that appeared unrealistic or did not correspond to respiratory motion from the evaluation. Based on the colour overlay assessment, the uncertainty of the registration results for the evaluation mask was within the pixel size. The results obtained in this study demonstrate that surrogate signals derived from 2D cine-MR images, including those generated by applying principal component analysis to the image intensities or control point displacements, can accurately model the internal motion as seen in 2D MR slices with both sagittal and coronal orientations. Future work will utilize the surrogate signals investigated in this paper for building 3D motion models following the approach of McClelland *et al* (2017), which can fit a 3D motion model directly to unsorted multi-slice 2D MRI data. However, validating the models and assessing their suitability for clinical use is challenging due to the difficulty in accurately estimating the true 3D motion.

In our recent and preliminary work (Tran *et al* 2019b) we built 3D motion models from multi-slice 2D MRI data with interleaved surrogate and motion slices using the approach of McClelland *et al* (2017). It took around 3 minutes to acquire 280 overlapping sagittal and coronal motion slices covering the thorax (i.e. 56 sagittal and coronal motion slices for 5 x 2-mm shifts). In these 3 minutes each point in space was sampled 10 times: 5 times with overlapping sagittal slices and 5 times with overlapping coronal slices. This would be comparable to the case of 10 training images in the current study, where we sampled each slice location 10 times obtaining mean DFEs around 1 mm for all 2-signal models except the one driven by the skin signal and derivative. However, due to the differences in the acquisition of the 2D and 3D MRI data, the results from investigating the effect of the number of images used to build the 2D models may not be directly applicable to 3D motion models. The general observations are likely to still apply, i.e.: that the internal motion can be modelled well with a relatively small number of training images (although the images used here represented multiple breath cycles and included both intra- and inter-cycle variation); that using more images to fit the models will improve the results, but the magnitude of the improvement will diminish and eventually plateau as more images are used; and that using too few images may result in over-fitting and reduced accuracy.

The datasets available for this study only had a total acquisition time of approximately one minute. Therefore, we were only able to assess the models over a relatively short amount of time. The accuracy of the models may decrease over time, either due to gradual changes in the breathing pattern, or sudden changes such as coughing. Future work will investigate the accuracy of the 3D motion models over longer periods of time which are comparable to the treatment time on an MR-Linac, and will develop methods to automatically estimate the accuracy of the 3D motion models, and to update or rebuild them if this is too low.

Furthermore, future work will utilize the 3D motion models for 4D dose calculations that accurately estimate the actual dose delivered during treatment, and for guiding treatment delivery on an MR-Linac.

## 5. Conclusions

In this paper we have evaluated several surrogate signals derived from 2D cine-MR images to model respiratory motion for lung cancer patients. We found that the signals generated by applying PCA to the control point displacements produced the highest model accuracy for sagittal slices and coronal slices (among the lowest errors). Also all other investigated signals were suitable to accurately model the respiratory motion of the internal anatomy within a single sagittal or coronal slice, with mean errors lower than the in-plane resolution. This implies the signals should also be suitable for modelling the 3D respiratory motion of the internal anatomy.
